# Safety and efficacy of combined epidural/general anesthesia during major abdominal surgery in patients with increased intracranial pressure: a cohort study

**DOI:** 10.1186/s12871-015-0056-2

**Published:** 2015-05-15

**Authors:** Igor Zabolotskikh, Nikita Trembach

**Affiliations:** Kuban State Medical University, Sedin st.,4, Krasnodar, 350063 Russian Federation

**Keywords:** Abdominal surgery, Intracranial pressure, Epidural anesthesia

## Abstract

**Background:**

The increased intracranial pressure can significantly complicate the perioperative period in major abdominal surgery, increasing the risk of complications, the length of recovery from the surgery, worsening the outcome. Epidural anesthesia has become a routine component of abdominal surgery, but its use in patients with increased intracranial pressure remains controversial. The goal of the study was to evaluate the safety and efficacy of epidural anesthesia, according to monitoring of intracranial pressure in patients with increased intracranial pressure.

**Methods:**

The study includes 65 surgical patients who were routinely undergone the major abdominal surgery under combined epidural/general anesthesia. Depending on the initial ICP all patients were divided into 2 groups: 1 (N group) - patients with the normal intracranial pressure (≤12 mm Hg, n = 35) and 2 (E group) – patients with the elevated intracranial pressure (ICP > 12 mm Hg, n = 30). During the surgery we evaluated ICP, blood pressure, cerebral perfusion pressure (CPP). The parameters of recovery from anesthesia and the effectiveness of postoperative analgesia were also assessed.

**Results:**

In N group ICP remained stable. In E group ICP decreased during anesthesia, the overall decline was 40 % at the end of the operation (from 15 to 9 mm Hg (P <0.05)). The correction of MAP with vasopressors to maintain normal CPP was required mainly in patients with increased ICP (70 % vs. 45 %, p <0.05). CPP declined by 19 % in N group. In E group the CPP reduction was 23 %, and then it remained stable at 60 mm Hg. No significant differences in time of the recovery of consciousness, effectiveness of postoperative analgesia and complications between patients with initially normal levels of ICP and patients with ICH were noted.

**Conclusions:**

The combination of general and epidural anesthesia is safe and effective in patients with increased intracranial pressure undergoing elective abdominal surgery under the condition of maintaining the arterial pressure. Its use is not associated with the increase in intracranial pressure during the anesthesia, but it needs an intraoperative monitoring of ICP in order to prevent CPP reduction.

## Background

Increased intracranial pressure (ICP) is a common state in surgical practice and it is mainly associated with the pathology of cerebral venous circulation, which significantly alters cerebral hemodynamics [1, 2]. Meanwhile, it can significantly complicate the perioperative period, increasing the risk of complications, the length of recovery from the surgery, worsening the outcome [1]. Total intravenous anesthesia with propofol and fentanyl worked well in the abdominal surgery, providing a smooth and rapid postoperative recovery, at the same time, this type of anesthesia is more preferable in the presence of increased ICP as compared with inhaled anesthetics [3, 4]. Epidural anesthesia has become a routine component of abdominal surgery because it provides a high quality of analgesia and promotes early mobilization of patients, including patients at high risk for perioperative complications. Nevertheless, according to some authors, it can lead to an increase in intracranial pressure in patients with reduced craniocerebral compliance [5]. An injection of both a local anesthetic and any solution may lead to an increase in ICP, which is associated with compression of the dural sac; thus, the increase is transient and not associated with properties of the local anesthetic [6]. Thereby, an increase in ICP was considered a contraindication to the use of epidural anesthesia over a long period of time, despite the small number of studies [7]. There is no conclusive evidence of a negative influence of epidural anesthesia on the perioperative period in patients with increased intracranial pressure, so, it makes this contraindication disputable, especially with a slow bolus injection of a local anesthetic, and all the more in case of its prolonged infusion [8]. Epidural anesthesia with 0.2 % ropivacaine solution administering as a continuous infusion has been successfully used in patients with a traumatic brain injury [9]. There are some reports about the safety and efficacy of epidural anesthesia in obstetric practice in patients with increased intracranial pressure [10]. As for the major abdominal surgery, where epidural anesthesia is almost the “gold standard” of anesthesia, its effect on ICP and the course of the perioperative period has not been studied well. Lack of investigations on this subject is due, primarily, to the fact that, despite its importance, the problem of evaluating of intracranial pressure in anesthesiology is currently far from being solved. Methods that we have in our disposal cannot be used routinely because these methods are either too invasive or not informative at all. In this regard, the method of ophtalmodynamometry (ODM) of the central retinal vein has an undoubted advantage. It allows us to perform a noninvasive and accurate evaluation of the intracranial pressure [11]. This method has been well proven in clinical practice [12].

Objective: To evaluate the safety and efficacy of epidural anesthesia, according to monitoring of intracranial pressure in patients with increased intracranial pressure.

## Methods

The paper presents the results of a cohort study conducted in 65 surgical patients (mean age 67 (65–77) years) who were undergone elective major abdominal surgery for cancer (hemihepatectomy, gastrectomy, hemicolectomy, duodeno-pancreatic resection) (mean duration of surgery - 7 (4–9) hours). Physical status was the 3 class according to American Society of Anesthesiologists (ASA) classification. Exclusion criteria were life-threatening decompensated severe systemic diseases, the 4–5 ASA class; massive intraoperative bleeding; alcohol and drug abuse;. The exclusion criteria were also contraindications for epidural anesthesia, that is, patient refusal, local infection, bleeding diathesis.

ICP was evaluated in all patients preoperatively using ODM of the central retinal vein in a horizontal position after local anesthesia of the sclera with 2 % lidocaine hydrochloride and pupil dilation with 0.5 % solution of mydriacyl. Depending on the initial ICP all patients were divided into 2 groups: 1 (N group) - patients with the normal intracranial pressure (≤12 mm Hg, n = 35) and 2 (E group) – patients with the elevated intracranial pressure (ICP > 12 mm Hg, n = 30) [13]. All patients had clear consciousness (Glasgow coma scale - 15 points).

All patients were NPO for at least 8 h before surgery and received 1 ml/kg normal saline (NPO deficit) per each NPO hour before induction as a part of fluid therapy in addition to the crystalloids used for the maintenance throughout the operation. All patients were placed on the operating table in a head up 15 degrees position, tracheal intubation in all cases was performed in a modified Jackson position. Introduction of anesthesia was performed in all groups with the following drugs: propofol (2 mg/kg), fentanyl (3 mkg/kg), non-depolarizing relaxant - atracurium (0.5 mg/kg). The infusion of propofol (6–12 mg/kg/h) was used in all patients to maintain anesthesia. The depth of anesthesia was controlled by the bispectral index, which was maintained at 40–60. An epidural catheterization was performed by a 18G Tuohy needle at Th8-Th10 before induction with the administration of 40 mg of lidocaine as a test dose. A bolus of 0.2 % solution of ropivacaine was injected to the epidural space (1 ml per spinal segment) followed by a continuous infusion (6–12 ml/h) for intraoperative analgesia. The infusion was continued postoperatively. Effectiveness of analgesia was assessed by a visual analogue scale (VAS) after awaking and then every 6 h for the first day. CPP was maintained at not less than 60 mm Hg. by the bolus administration of phenylephrine (25–50 mkg) or a continuous infusion of norepinephrine (0.8 % solution in a dose required to maintain arterial pressure, which usually does not exceed 0.1 mkg/kg/min). Mechanical ventilation was performed by Fabius or Julian (Draeger, Germany) and Blease Focus (Blease, UK) with the air-oxygen mixture (FiO_2_ – 0.4–0.5) to provide the normocapnia. The ventilation was corrected according to capnography data and arterial blood gas analysis with the 35–40 mm Hg target level of paCO_2_. All patients were actively warmed using a blowing warm air. The heart rate (HR, min-1), systolic (BPs, mm Hg) and diastolic (BPd mm Hg) blood pressure, mean arterial pressure (MAP, mm Hg) (Monitor Nihon Kohden, Japan) were among the studied haemodynamic parameters. The cerebral perfusion pressure (CPP) was calculated as the difference between the mean arterial pressure and intracranial pressure (CPP = MAP-ICP (mm Hg)). ICP measurement was performed on the following stages of anesthetic management: initially, after induction, and then every hour. The following parameters of recovery from anesthesia were evaluated postoperatively: time of consciousness recovery (the period from cessation of the anesthetic to spontaneous eye opening), time of full orientation recovery (the period from cessation of the anesthetic to the time when the patient can say the name and date of birth). A CAM-ICU scale was used to evaluate the patients for the purpose to diagnose delirium (daily until the patient’s discharge from the hospital). The incidence of adverse postoperative events, the length of stay in the ICU and in the hospital was also evaluated.

Statistics. Continuous data with normal distribution are given as mean ± standard deviation, otherwise as median (25–75 percentiles). The independent *t*-test for testing the significance of mean for independent continuous scale (of normal distribution) data, Mann–Whitney for the significance of mean for non-normal distribution data, Chi-squared or Fisher exact test for testing the significance of percentages (qualitative data) were used. A p value <0.05 was considered significant.

This study was approved by the ethics committee of KSMU, Krasnodar. All patients provided written informed consent.

## Results

There were no significant differences between the groups in body weight, age, and gender (Table 1).

**Table 1:** General characteristics of the patients

Parameter	Initial intracranial pressure	
	≤12 mm Hg	>12 mm Hg
Age (years)	67 (66–73)	69 (67–72)
Gender (% male)	56	60
Body mass index, kg/m^2^	27 (25–30)	24 (21–26)

The disturbances of venous cerebral blood flow determined by an transcranial Doppler, deferred stroke, head injury, encephalopathy, hypertension were more frequently observed in the E group. Otherwise, no significant differences were noted between the groups.

**Fig. 1:**
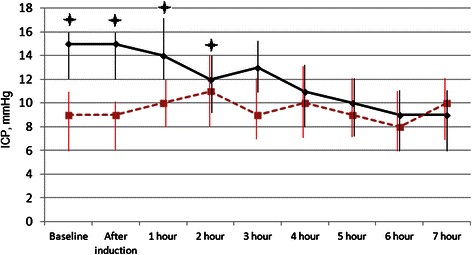
Dynamics of intracranial pressure (median with 25–75 percentile). In patients with initially normal levels of ICP, this value remained stable at all stages of the study. In the group with initially elevated ICP, it decreased during anesthesia. ICP – intracranial pressure. Dotted red line – group with normal initial ICP. solid black line - group with elevated initial ICP.  p <0.05 compared to patients with normal initial ICP

In N group patients, this value remained stable at all stages of the study. In the E group, it decreased during anesthesia, the overall decline was 40 % at the end of the operation (from 15 to 9 mm Hg (P <0.05)) (Fig. 1).

Baseline BP_S_ were significantly higher in the subgroups with initially increased ICP. All patients had the same dynamics, characterized by a decrease in BP_S_ to the third hour of the anesthesia with further stabilization. The reduction in BP_S_ within 14 % was observed in the subgroups with initially normal ICP. The reduction in BP_S_ was more pronounced and reached 25 % in the subgroups with increased ICP. The correction of MAP with vasopressors to maintain normal CPP was required mainly in patients with increased ICP (70 % vs. 45 %, p <0.05), which happened to a greater extent in the first 3 h of anesthesia until increased ICP was observed.

**Fig. 2:**
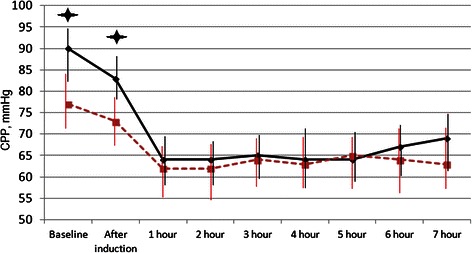
Dynamics of cerebral perfusion pressure (median with 25–75 percentile). In both groups, cerebral perfusion pressure decreased, but remained above 60 mm Hg. CPP – cerebral perfusion pressure. Dotted red line – group with normal initial ICP. solid black line - group with elevated initial ICP.  p <0.05 compared to patients with normal initial ICP

The analysis of the CPP showed its decline by 19 % in a N group with. In a E group, the CPP reduction was 23 %, and then remained stable at 60 mm Hg (Fig. 2).

No significant differences in time of the recovery of consciousness between patients with initially normal levels of ICP and patients with ICH were noted (Table 2). All patients were extubated within the first hour after the cessation of propofol infusion.

**Table 2:** Recovery time (Mean ± SD)

Registered parameter	Initial ICP ≤ 12 mm Hg	Initial ICP > 12 mmHg
Time of consciousness recovery, min	17.4 ± 6.3	19.3 ± 8.2
Time to full orientation, min	26.2 ± 9.5	28.6 ± 12.3

*ICP* – intracranial pressure

Effectiveness of epidural anesthesia was satisfactory in both groups at all stages of the study. There were no significant differences in pain intensity between the 2 groups (Table 3).

**Table 3:** Scores on the visual analogue scale (mean ± SD)

Stage	At rest		At movement	
	ICP ≤ 12 mm Hg	ICP >12 mm Hg	ICP ≤ 12 mm Hg	ICP >12 mm Hg
After waking up	1.4 ± 0.7	2 ± 0.8	2.3 ± 0.9	2.4 ± 1.3
After 2 h	3.3 ± 1.1	3.5 ± 1.4	3.8 ± 1.1	3.9 ± 1.2
After 8 h	3.1 ± 1.2	3.2 ± 1.1	3.3 ± 1.2	3.5 ± 0.9
After 24 h	2.3 ± 0.9	2.1 ± 1.0	2.8 ± 0.8	2.7 ± 1.1
After 2 days	2.1 ± 0.4	1.7 ± 0.6	2.5 ± 0.9	2.4 ± 0.9
After 3 days	1.2 ± 0.6	1.3 ± 0.7	1.8 ± 0.7	1.7 ± 0.7

*ICP* – intracranial pressure

The postoperative delirium (4 cases in N group and 3 cases in E group), pneumonia (3 cases in the N group and 2 cases in the E group) and arrhythmias (3 cases in N group and 1 case in E group) were the main perioperative complications. The lethal outcomes were not observed. All patients were transferred from the ICU and discharged from the hospital. There were no significant differences between the groups in the length of stay in the ICU (3,1 ± 1,1 days in N group vs 2,9 ± 1,5 days in E group) and in the hospital (14,5 ± 2,5 days in N group and 15 ± 2 days in E group).

## Discussion

Possibility of estimating of ICP has appeared at us as a result of the introduction of ophthalmodynamometry of central retinal vein. Over the past 10–15 years, several studies have confirmed the clinical relevance of ophthalmodynamometry in determining of intracranial pressure. Motschmann M., et al. showed a linear relationship between ICP and pressure in central retinal vein assessed invasively with a correlation coefficient of 0.968 [14]. So, authors concluded that the ophthalmodynamometry is valuable and accurate method of assessment of intracranial pressure, it can be used in patients with various neurological disorders such as hydrocephalus, brain tumor and head injury. Ophthalmodynamometry can be successfully used in the diagnosis of idiopathic intracranial hypertension [15]. A more recent study showed that the increase of pressure in central retinal vein in patients with idiopathic intracranial hypertension correlated with the direct measurement pressure of cerebrospinal fluid through a lumbar puncture [16]. In 2010, a group of researchers conducted a blind research of ophthalmodynamometry in neurological hospital patients and healthy volunteers at high altitudes [17]. Ophthalmodynamometry showed a significant correlation with ICP determined by a ventricular sensor. The correlation coefficient was 0.85, the accuracy of the method - 89 %. In 2011, a group of German collaborators published a paper on the definition of accuracy of ophthalmodynamometry in the evaluation of intracranial pressure. The study included 102 patients with diseases of the brain. Ophthalmodynamometry has proved to be an accurate noninvasive method for the diagnosis of intracranial hypertension with a sensitivity of 84.2 % and a specificity of 92.8 % [11]. The main limitation of this method is its discrete nature. Thus, it allows us to estimate only the trend, abrupt changes in intracranial pressure may remain unnoticed.

We observed a moderate decrease in intracranial pressure during anesthesia with propofol which is consistent with the work carried out earlier showed that propofol significantly reduces ICP in patients with intracranial hypertension. Such dynamics is connected with the fact that propofol reduces the cerebral metabolic rate [18, 19], and also causes cerebral vasoconstriction and proportional decrease in cerebral blood flow [20]. There are a number of publications, showing an increase of this indicator on application of epidural anesthesia [21–23], which is more pronounced in patients with an initial tendency to intracranial hypertension [6]. It is primarily resulted from the dural sac external compression reducing its capacity [5]. These changes can lead to the inadequate epidural anesthesia and make complications more frequent [24]. According to our data, no increase in intracranial pressure with epidural anesthesia, and no effect of the elevated ICP on the efficiency of epidural anesthesia can be explained by methodological differences between researchers. The bolus injection of the main dose of the local anesthetic solution was used in all studies that showed an increase in intracranial pressure whereas we used a method of the continuous infusion in our research. This is supported by the studies claiming that the epidural anesthesia with the infusion administration of solutions with the low concentration of local anesthetics is safe in patients with increased intracranial pressure [9, 24]. BP_S_ was significantly reduced, which is specific for a sympathetic blockade and vasodilation inherent to propofol, however, the simultaneous reduction of intracranial pressure allowed us to maintain CPP at a safe level in all cases. However, it should be noted that maintaining a minimum safe level of CPP at 60 mm Hg [25, 26], required more frequent use of vasopressors in patients with initially elevated ICP. Thus, in the absence of monitoring of intracranial pressure in patients with elevated ICP there is a risk of reduction of CPP below a safe level even at normal BP_S_.

## Conclusion

The combination of general and epidural anesthesia is safe and effective in patients with increased intracranial pressure undergoing elective abdominal surgery under the condition of maintaining the arterial pressure. Its use is not associated with the increase in intracranial pressure during the anesthesia, but it needs an intraoperative monitoring of ICP in order to prevent CPP reduction due to a hypotension inherent to EA.

## Abbreviations

ICP: Intracranial pressure
CPP: Cerebral perfusion pressure
MAP: Mean arterial pressure
ODM: Ophtalmodynamometry
NPO: Nothing per os
VAS: Visual analogue scale
HR: Heart rate
BPs: Systolic blood pressure
BPd: Diastolic blood pressure
KSMA: Kuban State Medical University
